# Selenium Nanoparticles as Versatile Delivery Tools

**DOI:** 10.3390/pharmaceutics17121556

**Published:** 2025-12-03

**Authors:** Amir Nasrolahi Shirazi, Rajesh Vadlapatla, Ajoy Koomer, Kyle Yep, Keykavous Parang

**Affiliations:** 1 Department of Pharmaceutical Sciences, College of Pharmacy, Marshall B. Ketchum University, 2575 Yorba Linda Blvd., Fullerton, CA 92831, USA; rvadlapatla@ketchum.edu (R.V.); akoomer@ketchum.edu (A.K.); kyleyep.cop28@ketchum.edu (K.Y.); 2 Center for Targeted Drug Delivery, Department of Biomedical and Pharmaceutical Sciences, Chapman University School of Pharmacy, Harry and Diane Rinker Health Science Campus, 9401 Jeronimo Rd, Irvine, CA 92618, USA; parang@chapman.edu

**Keywords:** selenium nanoparticles, drug delivery, metal nanoparticles, anticancer activity

## Abstract

Selenium nanoparticles (SeNPs) have emerged as promising metal-based nanoparticles for drug delivery due to their unique physicochemical properties, intrinsic bioactivity, and biocompatibility. SeNPs offer a lower toxicity, higher bioavailability, and flexibility to be customized for surface chemistry compared to traditional selenium compounds. Advances in synthetic strategies, including chemical reduction, green biosynthesis, and surface functionalization with polymers, peptides, or ligands, have improved their stability, targeting capability, and circulation time. SeNP-based systems have demonstrated unique anticancer, antimicrobial, and anti-inflammatory activities, as they can function as drug carriers and active therapeutic agents. The surface of SeNPs has been functionalized with ligands such as Arginylglycylaspartic acid (RGD) peptides, hyaluronic acid, or chitosan to enhance their receptor-mediated targeting abilities in tumor tissues. In addition, SeNPs have shown a synergistic effect in the presence of drugs such as doxorubicin and paclitaxel. Even though SeNPs have demonstrated significant potential in pre-clinical investigations, their use in clinical studies has not been expanded due to several limiting challenges, including large-scale production, long-term safety, pharmacokinetic properties, and regulations required for FDA approval. Continued research into optimizing formulation strategies and expanding in vivo validation will be critical to translating SeNP-based drug delivery systems into clinical applications. In this review, we focus on the methods for synthesizing SeNPs, their physicochemical properties, the structure of ligands attached to SeNPs for drug delivery applications, and the specific biological targets of functionalized SeNPs.

## 1. Introduction

### 1.1. The Metal Nanoparticles in Drug Delivery

Metal nanoparticles (MNPs) have garnered considerable attention within the field of nanomaterials due to their unique size-dependent properties and versatile functionalities. Due to their customizable size, reactive surface area, multifunctionality, including the ability to target and image simultaneously, and potential for controlled release, MNPs offer numerous advantages over other materials for drug delivery [1]. Commonly studied MNPs include gold nanoparticles (AuNPs), silver nanoparticles (AgNPs), iron oxide nanoparticles (Fe_3_O_4_ NPs), platinum nanoparticles (Pt NPs), zinc oxide nanoparticles (ZnO NPs), selenium nanoparticles (SeNPs), and gadolinium nanoparticles (GdNPs), which can be synthesized via chemical, physical, or biological methods. These nanoparticles have been extensively studied for their unique properties, which are applicable to both therapeutic and diagnostic purposes [2].

AuNPs and AgNPs have demonstrated optimized surface plasmon resonance (SPR), which makes them a prominent tool in drug delivery and imaging [3]. Although they offer an extensive set of advantages in drug delivery, their full utilization is hindered by challenges such as cytotoxicity, low stability, and difficulties in large-scale production [4].

AuNPs are highly valued for their stability, biocompatibility, and surface plasma resonance (SPR) properties. They can be readily functionalized with drugs, peptides, or targeting ligands for tumor-selective delivery and are widely utilized in photothermal therapy and imaging [5]. Functionalized AuNPs enhance drug solubility, increase tumor accumulation, and reduce systemic side effects [6].

AgNPs exhibit potent antimicrobial activity, supporting wound healing and the management of infectious diseases. Additionally, their anti-inflammatory and anticancer effects mediated via reactive oxygen species (ROS) generation and membrane disruption enable synergistic drug delivery when co-loaded with antibiotics or chemotherapeutics [7].

Fe_3_O_4_ NPs possess superparamagnetic behavior, facilitating magnetic targeting and magnetic resonance imaging (MRI) contrast enhancement. Coating with polymers or peptides allows precise drug delivery to tumors or inflamed tissues under external magnetic guidance [8]. Their biocompatibility and biodegradability make them suitable for multifunctional theranostic applications, including improved cellular uptake and controlled release of drugs such as 5-fluorouracil (5-FU) [9].

ZnO NPs function as pH-sensitive carriers, disintegrating into acidic tumor environments to promote localized drug release and ROS-mediated cytotoxicity, highlighting their potential in cancer therapy [10]. GdNPs, primarily recognized as MRI contrast agents due to gadolinium’s paramagnetic properties, are increasingly engineered as theranostic platforms that integrate diagnostic imaging with drug delivery or photothermal therapy [11]. Their dual functionality enables precise monitoring and treatment, particularly in oncology.

Although metal nanoparticles display highly varied properties and application-specific strengths, SeNPs have recently attracted attention for their antioxidant, anticancer, and immunomodulatory properties [12]. They inhibit tumor proliferation through the induction of apoptosis and redox modulation while exhibiting minimal toxicity toward normal cells [13]. Functionalization with targeting ligands or drugs enhances their specificity and therapeutic efficacy, making them promising vehicles for delivering chemotherapeutics and natural compounds in the treatment of cancer, cardiovascular, and inflammatory disorders [14]. We have previously reported a number of functionalized MNPs, including SeNPs, as multifunctional platforms with the potential to greatly enhance future drug delivery and diagnostic applications [15,16,17,18,19]. This review provides an integrated overview of the role of selenium in our body, applications of SeNPs, covering their synthesis methods, fundamental physicochemical characteristics, the design and structure of ligands used to functionalize SeNPs for drug delivery, the biological targets to which they are engineered to bind, challenges and limitations, and future perspectives.

### 1.2. Selenium

**Figure 1:**
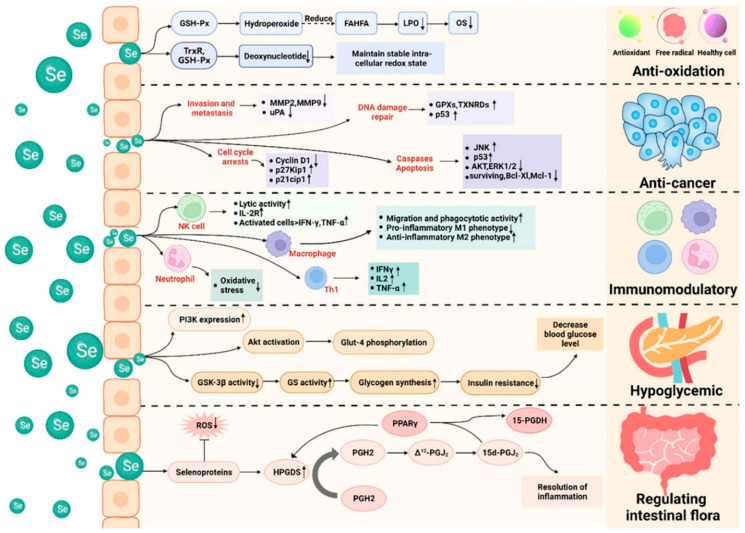
Health-promoting effects of selenium and its mechanisms of action. Selenium possesses anti-oxidant, anti-cancer, immunomodulatory, hypoglycemic, and intestinal microbiota-regulating properties. It acts as an antioxidant by lowering oxidative stress and deoxynucleotide levels. It functions as an anti-cancer agent by inducing apoptosis and cell-cycle arrest, preventing tumor cell invasion and metastasis, and promoting DNA damage repair. It exerts immunomodulatory effects by affecting non-specific immunity (e.g., macrophages and neutrophils) and specific immunity (e.g., T and B lymphocytes). It exerts hypoglycemic action by regulating the IRS-PI3K-Akt signaling pathway. It regulates the intestinal microbiota by regulating prostaglandins. This Figure was reproduced from reference [29] by Sun et al. under the terms of the Creative Commons Attribution (CC BY) license.

Selenium (Se) is an essential trace element required for optimal human growth, development, and physiological homeostasis [20]. In humans, selenium exists in several chemical forms, including inorganic species (selenate, SeO_4_^2−^; selenite, SeO_3_^2−^) and organic forms (selenomethionine and selenocysteine). Dietary selenium is primarily absorbed in the duodenum and proximal jejunum through both active and passive transport processes, with organic selenium forms generally exhibiting higher bioavailability. Once absorbed, selenium undergoes metabolic conversion to selenide (HSe^−^), which serves as a precursor for the biosynthesis of selenocysteine, the 21st amino acid, through a unique co-translational incorporation mechanism mediated by the selenocysteine insertion sequence (SECIS) element in messenger RNA (mRNA). This process enables selenium to be incorporated into at least 25 known human selenoproteins, which collectively mediate antioxidant defense, redox signaling, thyroid hormone activation, and immune regulation [21]. Selenium metabolism and biosynthesis of selenoproteins in the human body have been investigated [22]. The role of selenium metabolism and selenoproteins in cartilage homeostasis and arthropathies [22]. Here are a few examples of selenium’s impact:

Selenium’s role in antioxidant defense and redox homeostasis is significant. It is incorporated into selenoproteins such as glutathione peroxidases (GPx), thioredoxin reductases (TrxR), and other redox enzymes. These detoxify reactive oxygen species (ROS), reduce lipid peroxides, and maintain cellular redox balance [23,24]. The role of selenium in maintaining proper thyroid activity and the normal functioning of the immune system reiterates the importance of maintaining adequate intake levels to support these processes. Nonetheless, excessive intake may cause contraindications, such as selenium toxicity. Therefore, balancing selenium intake to maintain its optimal level through a balanced diet and cautious supplementation is essential for maintaining health [25]. In addition, it has been utilized in the management of conditions such as human immunodeficiency virus (HIV) [26], inflammatory bowel disease (IBD) [27], cardiovascular disease (CVD) [28], and various types of cancer. Common forms of supplementation include inorganic sodium selenite, organic selenium compounds, selenium-enriched yeast, and SeNPs. The relationship between selenium and human health is complex, showing a U-shaped dose–response pattern in which deficiency benefits from supplementation, while excess causes harm. Due to the limited safety margin and the baseline levels varying across different populations, establishing an optimal intake and safe supplementation remains critical for future research (Figure 1) [29].

Selenium’s role in immune function and inflammation regulation is proven. Studies showed that adequate selenium supports both innate and adaptive immunity [11]. It modulates cytokine production, influences lymphocyte proliferation, and can help regulate inflammatory pathways. A recent epidemiological review found that selenium intake correlates with better outcomes in infectious disease settings and with modulation of immune function. Additionally, the impact of selenium intake is critical for regulating the immune system [29]. Several recent studies have found that adequate selenium status appears to be associated with better immune outcomes in conditions such as infections, including COVID-19, influenza, and hepatitis, demonstrating its immunoregulatory impact. Additionally, selenium has been shown to influence the nuclear factor erythroid 2-related factor 2 and nuclear factor-kappa B signaling pathways. As a result, it performs the antioxidant and anti-inflammatory roles. These properties allow selenium to maintain endothelial activity, drive the development of atherosclerosis, and provide defense against chronic inflammatory illnesses [12,30]. At the same time, the outcomes of a research study based on the Newcastle 85+ longitudinal cohort revealed that higher baseline plasma selenium and selenoprotein P concentrations were associated with better baseline cognitive performance; however, no significant association with the long-term cognitive trajectory has been found. Consequently, selenium physiological volume may help maintain, not change, older people’s cognitive function [31].

Selenium is an essential element in regulating thyroid hormone metabolism and autoimmunity. The thyroid gland has one of the highest concentrations of selenium, due to the reliance on selenoproteins (e.g., iodothyronine deiodinases) for hormone activation and antioxidant protection during hormone synthesis [32]. Selenium supplementation in patients with autoimmune thyroiditis (e.g., Hashimoto’s disease) has been associated with reductions in anti-thyroid peroxidase (TPO) antibody titers and improvements in antibody levels over 6–12 months [33]. In addition, a 2025 clinical trial showed that selenium (especially selenomethionine) reduced thyroid peroxidase antibodies (TPOAb) and thyroid-stimulating hormone (TSH) in Hashimoto’s patients, improving mood/well-being [23]. However, not all trials find benefit. Another study found that selenium supplementation and placebo were equally effective in improving quality of life in hypothyroid patients [34].

Selenium may offer benefits for cardiovascular health and be associated with improving mortality outcomes. Studies show that Selenium may protect against endothelial dysfunction, oxidative damage in vascular tissues, and atherosclerosis. Cui et al. conducted a meta-analysis investigation and found that biomarkers of selenium status were inversely associated with all-cause, cardiovascular, and cancer mortality in population cohorts [35]. However, the U.S. Office of Dietary Supplements notes that clinical trial evidence in healthy individuals does not support the use of selenium supplementation to reduce cardiovascular events [36]. One large, randomized trial (SELECT) and other trials found no significant reduction in cardiovascular events with selenium supplementation alone (100–800 µg/day) in populations that were mostly selenium-replete [36].

It has been proposed that selenium can prevent cancer or enhance chemotherapy outcomes in cancer patients. Selenium has been proposed to reduce cancer risk via antioxidant, anti-proliferative, apoptotic, and DNA repair pathways [37]. According to the report by Wang et al., higher selenium intake was associated with a reduced risk of certain digestive system cancers [38]. However, evidence is mixed, and many studies are observational with low quality, making causal inference difficult.

Selenium plays an important role in Reproductive Health. Selenium influences male fertility (sperm motility, mitochondrial sheath integrity) and is implicated in female reproductive function (e.g., ovarian health) [39]. Bai et al. emphasized the importance of selenium in reproductive health. In the male reproductive system, selenium contributes to spermatogenesis through its presence in phospholipid hydroperoxide glutathione peroxidase (PHGPx), which stabilizes sperm mitochondrial membranes [25]. It is important to mention that selenium deficiency can lead to Keshan disease (a cardiomyopathy endemic in low-selenium soils) and is associated with increased mortality and disease risk in some populations [38]. Toxicity (selenosis) can occur at high intake (>330–450 µg/day in some reports) and cause symptoms such as gastrointestinal upset, hair/nail loss, neuropathy, fatigue, and even severe effects on nervous and cardiac systems [40]. In addition, the therapeutic window between beneficial and harmful is relatively narrow, so context (baseline status, region, diet, comorbidities) matters [25,41]. Taken together, the accumulating clinical and epidemiological evidence demonstrates the irreplaceable importance of selenium in human health and its role in preventing chronic disease. These findings have stimulated extensive mechanistic research to elucidate the mechanisms by which selenium’s protective effects operate at the molecular and cellular level. This is a rapidly developing area, and the growing knowledge of selenium’s molecular biology not only describes its many physiological functions but also permits the rational design of new Se-based therapeutic interventions and supplementation recommendations to maximize health benefits while minimizing the risk of toxicity.

### 1.3. Selenium Nanoparticles

An emerging class of nanomaterials, selenium-based nanoparticles, has attracted significant interest due to their multi-dimensional applications in biomedicine. Generally, SeNPs exhibit bioavailability and biodistribution, low toxicity, and slow release of selenium, thereby reducing the risk of selenosis and retaining the therapeutic potential of selenium [42]. The chemical and physical properties of nanoparticles make them ideal for interacting with biological systems, including the host-microbe-parasite interface, and give them capabilities not inherent in bulk materials. SeNPS also scavenges ROS and enables redox balance by enhancing the function of selenoenzymes such as GSH-Px and Thioredoxin reductase (TR) [43]. Thioredoxin reductase (TR) is a selenoprotein enzyme that works together with thioredoxin (Trx) to maintain the cellular redox balance. It reduces oxidized thioredoxin using NADPH, thereby supporting antioxidant defense systems, DNA synthesis, and cell survival in the presence of oxidative stress. Due to these properties, SeNPs can protect cells and cellular structure from exposure to oxidative stress.

In recent years, SeNPs have attracted significant attention as multi-functional vehicles for cancer therapy and drug delivery. The dual capability of SeNPs, to induce oxidative stress and apoptosis in most cancer cells while having minimal effect on normal cells, centers their use in cancer therapy. Moreover, SeNPs have been adapted for other applications, such as neuroprotection [44], antimicrobial drugs [45], and wound healing, due to their unique properties and biocompatibility. Continued research on green synthesis methods, pharmacokinetics, and toxicity mechanisms will be crucial to advance SeNPs from laboratory investigations toward clinical translation and therapeutic applications [46].

It should be noted that selenium is an essential micronutrient with a recommended dietary intake of 55 μg/day [47]. Although selenium is required for normal cellular function, high doses of selenium in the form of SeNPs could selectively kill malignant cells [48]. Thus, there is a compelling need to design novel SeNPs that could be used as nontoxic nano-drug delivery systems (DDSs). Therefore, two parameters, including the chemical entity of selenium and concentration, are responsible for its toxicity and anticancer activity. Selenium can be found in different oxidation states, namely, selenite (SeO_3_^2−^) oxyanions, selenite (SeO_4_^2−^), and elemental selenium (Se^0^), with oxidation states of +4, +6, and 0, respectively. It is worth mentioning that, as with the majority of metal ions, the toxicity of these metal ions can be reduced or eliminated by altering their redox state [49]. In the next section, we discuss the ligand systems used to prepare and functionalize SeNPs for biomedical applications.

#### 1.3.1. Ligands

Ligands that bind to SeNPs play a crucial role in determining their function, stability, and transportation capabilities. Ligands are divided into several categories based on their nature, including polymers, peptides and proteins, nucleic acids, small molecules, natural products, polysaccharides, and lipids. Here is a list of ligands that were used in conjugation with SeNPs.

##### Polymers

Polymers offer stability and controlled release, and they are the most widely used ligands for SeNPs conjugation because they improve colloidal stability, prevent aggregation, and enable sustained drug release.

**Chitosan (Cs):** Chitosan is a cationic, biocompatible polysaccharide that enhances mucosal adhesion and cellular uptake. Chitosan-coated SeNPs improve stability and enable loading of negatively charged drugs (e.g., doxorubicin, curcumin). Al-Darwesh et al. synthesized functionalized SeNPs from sodium selenite (Na_2_SeO_3_) using *Foeniculum vulgare* seed extract as a mild and green reducing agent. The reaction was carried out at 30 °C for 60 min, yielding 87% of red SeNPs, which were stabilized in phosphate-buffered saline (PBS, pH 7.4). Chitosan-coated SeNPs were subsequently prepared by adding a chitosan–glacial acetic acid mixture to the SeNPs. Paclitaxel (PTX) was then loaded onto the chitosan-coated SeNPs by stirring them in a PTX–DMSO solution. The final chitosan-coated SeNPs-paclitaxel were obtained in a yield of 0.8 g and could be stably stored in de-aerated PBS (pH 7.4) under light-protected conditions for at least one week [50], demonstrating the feasibility of this eco-friendly method for developing stable nanocarriers for drug delivery.

Cetin et al. developed a multi-step approach for producing chitosan-coated, ferulic-acid-loaded SeNPs. In their method, ferulic acid was first incorporated into SeNPs formed by reducing sodium selenite in the presence of a stabilizing agent, followed by purification to remove unreacted materials. The resulting ferulic-acid-loaded SeNPs were subsequently coated with chitosan to improve stability and functional performance [51].

For surface functionalization, alginate and chitosan-coated ferulic-acid-loaded SeNPs were prepared. To enhance surface stability and functionality, the authors produced alginate- and chitosan-coated ferulic-acid-loaded SeNPs. Alginate coating was achieved by combining ferulic-acid-loaded SeNPs with an alginate solution and facilitating polymer adsorption through brief sonication and mixing. Subsequent calcium-mediated cross-linking stabilized the coating, and the resulting nanoparticles were collected and washed before resuspension [51]. Ferulic acid-loaded SeNPs coated with alginate or chitosan exhibited dose-dependent cytotoxicity against the Triple-negative breast cancer (TNBC) cell line MDA-MB-231, with the alginate–coated variant showing stronger potency (IC_50_ = 103.6 µg/mL) than the chitosan–coated (IC_50_ = 178.7 µg/mL). Biomarker analyses indicated genotoxic stress and apoptotic pathways were engaged.

Dana et al. developed chitosan-coated SeNPs to enhance the stability of SeNPs and assess their activity toward glioma cells. SeNPs were produced via the reduction of sodium selenite with L-ascorbic acid, followed by purification to remove unreacted components. The chitosan-coated formulation was prepared using the same reduction approach, with chitosan incorporated during synthesis to form a stabilizing polymer shell. The resulting nanoparticles were purified and subsequently characterized for selenium content, size, and surface charge. The chitosan coating conferred improved physical stability: the Cs-SeNPs showed more consistent hydrodynamic diameter and zeta potential over 14–15 days at 4 °C, compared with uncoated SeNPs [52]. The study found that chitosan-coated SeNPs show superior anticancer activity against glioma cells compared to uncoated SeNPs. In a monolayer culture of the glioma cell line U87, both SeNPs and Cs-SeNPs reduced cell viability in a dose-dependent manner. However, Cs-SeNPs exhibited stronger selectivity: at the optimal chitosan coating (0.2% chitosan), the reduction in glioma cell viability was greater while toxicity to normal fibroblasts was lower. They decrease viability, inhibit spheroid growth, impair migration/invasion, enhance uptake, cross a BBB model, and increase sensitivity to 5-FU, while also being more stable physically. This makes Cs-SeNPs a promising nanoplatform for glioma/brain cancer therapy, albeit still in the preclinical stage.

Dana et al. also prepared coumarin-6-loaded chitosan-coated SeNPs by introducing coumarin-6 during the nanoparticle formation step. In the chitosan-coated version, chitosan was pre-mixed with water, followed by the addition of sodium selenate, coumarin-6, and reducing agent, allowing both nanoparticle formation and surface coating to occur simultaneously in a single process. The labeled nanoparticles were then purified using the established dialysis protocol [52]. U87 glioma cells internalized chitosan-coated coumarin-6 SeNPs significantly more efficiently than uncoated SeNPs, with fluorescence intensity 9.2-fold higher at 0.5 h, 2.9-fold at 1 h, and 2.4-fold at 2 h post-exposure.

**Polyethylene glycol (PEG):** PEG is a polymer that provides a stealth coating that prolongs circulation time and reduces recognition by the reticuloendothelial system (RES). Polyethylene glycolated SeNPs are commonly used for the systemic delivery of drugs. Mary et al. reported a modified method for the synthesis of polyethylene glycol-SeNPs using a previously reported procedure with minor modifications [53,54]. In this approach, polyethylene glycol-SeNPs were generated by thermally treating sodium selenite with PEG at elevated temperature (210–220 °C), allowing reduction and nanoparticle formation to occur simultaneously. The resulting colloid was subsequently diluted, collected, and washed to eliminate residual polymer. The finalized polyethylene glycol-SeNPs were analyzed using various spectroscopic techniques [53]. The polyethylene glycol-SeNPs–Crocin conjugate exhibited enhanced cytotoxicity against lung cancer cells (A549) compared with crocin alone. In vivo, treatment with the polyethylene glycol-SeNPs–Crocin formulation significantly inhibited tumor growth in a nude mouse model compared to controls, supporting the improved anticancer effect via synergism.

**Polyvinylpyrrolidone (PVP):** PVP is used to stabilize SeNPs during synthesis and enhance biocompatibility. Zhu et al. synthesized polyvinylpyrrolidone (PVP)-Glucose-SeNPs-loaded-doxorubicin using an ascorbic acid-mediated reduction of sodium selenite in the presence of PVP and glucose, allowing simultaneous nanoparticle formation and surface functionalization (PVP = P and Glucose = G). As the reaction progressed, the solution gradually shifted from colorless to orange and then red, indicating successful SeNP formation. Characterization revealed that polyvinylpyrrolidone (PVP)-Glucose-SeNPs were produced via the ascorbic acid-mediated reduction of sodium selenite and co-functionalized with PVP and glucose. The absorption band at 1661 cm^−1^, attributed to the C=O stretching vibration of PVP, appeared in PVP, polyvinylpyrrolidone (PVP)-Glucose-SeNPs, and polyvinylpyrrolidone (PVP)-Glucose-SeNPs-loaded-doxorubicin, confirming successful PVP coating on the nanoparticle surface [54]. The doxorubicin-loaded PVP-Glucose-SeNPs exhibited enhanced antitumor activity in breast cancer models, demonstrating strong synergy in cytotoxicity.

**Polylactic-co-glycolic acid (PLGA):** PLGA is a biodegradable polymer allowing controlled drug release and improved pharmacokinetics. Al-Shreefy et al. reported a method for the synthesis of SeNPs and encapsulated them into PLGA polymer [55]. SeNPs were synthesized by reducing sodium selenite with ascorbic acid in the presence of a water-soluble derivative of natural vitamin E (D-α-Tocopheryl Polyethylene Glycol 1000 Succinate, TPGS), as a stabilizer. The reaction mixture changed from colorless to orange-red, indicating nanoparticle formation, and was stirred at room temperature for 24 h. The resulting Se- D-α-tocopheryl polyethylene glycol 1000 succinate nanoparticles were purified by dialysis to remove unreacted components.

For encapsulation, SeNPs were incorporated into polylactic-co-glycolic acid (PLGA) using an emulsion solvent evaporation technique. PLGA dissolved in dichloromethane (DCM) was mixed with the Se-D-α-tocopheryl polyethylene glycol 1000 succinate solution, sonicated, and stirred to evaporate DCM. The obtained SeNPs- polylactic-co-glycolic acid nanocomposites were washed, centrifuged, and dried.

Similarly, SeNPs were encapsulated into polylactic-co-glycolic acid–polyethylene glycol–folic acid (folic acid-modified PLGA-PEG) using a double emulsion solvent evaporation method. The SeNP solution was emulsified with polylactic-co-glycolic acid–polyethylene glycol–folic acid in DCM, followed by a second emulsification with polyvinyl alcohol (PVA). After solvent evaporation, the nanoparticles were purified by sequential centrifugation and resuspension in deionized water [55]. PLGA-encapsulated SeNPs, including folate-modified versions (PLGA–PEG–FA), exhibited strong cytotoxic effects against MCF-7 breast cancer cells while maintaining low toxicity toward normal HBL-100 breast epithelial cells, indicating selective anticancer activity. The formulations induced apoptosis (confirmed by AO/EB staining), exhibited antioxidant activity in 2,2-diphenyl-1-picrylhydrazyl (DPPH) colorimetric assays, and demonstrated good hemocompatibility, with hemolysis levels within safe limits. Folate-functionalized particles further enhanced uptake and cytotoxicity in MCF-7 cells.

**Mesoporous silica:** Mesoporous silica-coated SeNPs were fabricated through a multi-step synthesis process. Initially, SeNPs capped with polyvinylpyrrolidone (PVP) were produced, exhibiting a diameter of approximately 90 nm and a surface charge of −4 mV. These particles were then coated with a silica shell via hydrolysis of (3-aminopropyl)triethoxysilane (APTES), forming SeNP@nSiO_2_ core–shell structures about 110 nm in size. A subsequent Cation-active cetyltrimethylammonium bromide (CTAB)-templated condensation of tetraethyl orthosilicate (TEOS) generated an additional mesoporous silica layer, yielding SeNP@nSiO_2_@mSiO_2_ nanocarriers with a final diameter of ~125 nm and a ζ-potential of −8.5 mV. Dynamic light scattering (DLS) analysis confirmed the size distribution observed by TEM [56]. When loaded with doxorubicin, these nanoparticles demonstrated synergistic cytotoxicity against tumor cells. The system featured acid-responsive folate exposure, enhancing tumor-specific targeting and cellular uptake under mildly acidic tumor microenvironments. This multi-layered nanocarrier enabled programmed drug release and improved anticancer efficacy.

##### Peptides

Peptides are excellent targeting ligands due to their high specificity in receptor affinity and biodegradability.

**Figure 2:**
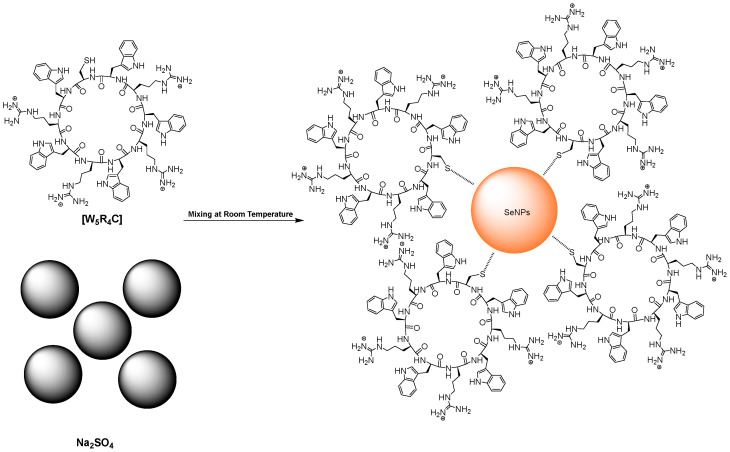
A schematic demonstration of the synthesis of [Trp-Arg-Trp-Arg-Trp-Arg-Trp-Arg-Trp-Cys]-SeNPs drug delivery systems. Guanidine groups in the structure of arginine amino acids are partially positively charged in aqueous media.

**Peptides containing arginine, tryptophan, and cysteine [Trp-Arg-Trp-Arg-Trp-Arg-Trp-Arg-Trp-Cys]**. Peptides containing arginine (R), tryptophan (W), and cysteine (C) were found to be an excellent ligand/reducing agent for selenium particles. Through one-pot synthesis, a selenium oxide solution can be converted to SeNPs, which is observable by a change in color. At the same time, the peptide functions as a ligand by capping the SeNPs and stabilizing them in an aqueous solution. The results showed that the molecular transporting ability of the peptide remains intact upon formation of SeNPs (Figure 2) [57]. Furthermore, [Trp-Arg-Trp-Arg-Trp-Arg-Trp-Arg-Trp-Cys]-SeNPs were able to load doxorubicin as a drug cargo and enhance its intracellular uptake and antiproliferative activity. In addition, peptide-containing arginine and tryptophan-functionalized SeNPs were used as effective delivery systems for Src-targeting siRNA in triple-negative breast cancer cells by Suryakanta et al. [58].

**RGD peptide (Arg–Gly–Asp):** RGD targets integrin αvβ3 receptors overexpressed in the tumor vasculature and many cancer cells. RGD-functionalized SeNPs enable tumor-specific accumulation and improved cytotoxicity toward malignant tissues. Fu et al. reported a method for the synthesis of RGD peptide-conjugated SeNPs [59].

The chitosan-RGD conjugate was generated by coupling the RGD peptide to chitosan through a 3-maleimide propionic acid-*N*-hydroxysuccinimide (maleimide–NHS) ester-mediated reaction, allowing stable peptide attachment to the polymer. The resulting conjugate was isolated and dried for use in nanoparticle functionalization. RGD-functionalized SeNPs were then prepared by reducing sodium selenite with ascorbic acid in the presence of doxorubicin, enabling simultaneous nanoparticle formation and drug incorporation. As the reduction proceeded, elemental selenium nucleated and assembled into SeNPs. RGD-functionalized SeNPs loaded with doxorubicin exhibited enhanced tumor targeting and antiangiogenic activity. In vitro, the RGD-SeNPs significantly inhibited proliferation and induced apoptosis in HUVECs, suppressed VEGF-induced tube formation, and disrupted angiogenesis by downregulating the VEGF-VEGFR2-ERK/AKT signaling pathway. In vivo, in an MCF-7 xenograft mouse model, the nanoparticles resulted in a marked reduction in tumor volume and microvessel density, accompanied by lower expression of VEGF and VEGFR2 in tumor tissues.

**Angiopep-2:** Angiopep-2 is used for targeted therapy of glioma in combination with functionalized SeNPs [60]. The functionalized SeNPs were synthesized using a multi-step process. Mesoporous SeNPs (MSe) were first prepared with mesoporous silica as a hard template and sodium selenite as the selenium source. PTX was then loaded into Mesoporous SeNPs via adsorption to form MSeNPs. The particles were coated with a phospholipid layer using thin-film dispersion. Separately, an Angiopep-2-functionalized C6 cell membrane was obtained by forming amide bonds between Ang-2 carboxyl groups and amino groups on membrane surface proteins. This functionalized membrane was fused with lipid-coated MSeP to produce ACMLMSeP (Ang2-modified cell membranes (ACM). The resulting MSe core enhances reactive oxygen species (ROS) generation in tumor cells, inducing apoptosis and autophagy. Angiopep-2-functionalized mesoporous SeNPs (ACMLMSeP), loaded with paclitaxel and coated with glioma-cell-derived membranes, demonstrated enhanced targeting and therapeutic efficacy against glioma. The Angiopep-2 ligand facilitated efficient crossing of the blood–brain barrier and selective accumulation in glioma tissue through LRP receptor targeting. Biologically, the nanoparticles induced potent apoptosis and autophagy through dual mechanisms: paclitaxel-mediated cytotoxicity and reactive oxygen species (ROS) generation from the selenium core.

##### Small Molecule Ligands

Small molecules or biomolecules are often conjugated to SeNPs to confer targeting specificity or therapeutic synergy.

**Folic acid (FA):** Folic acid targets folate receptors overexpressed on various cancer cells (e.g., ovarian, breast, and cervical). Folic acid-SeNPs deliver anticancer drugs with enhanced selectivity and uptake. SeNPs were synthesized by reducing selenium dioxide (SeO_2_) with ascorbic acid under stirring, followed by centrifugation and incubation in the dark to form stable nanoparticles. Curcumin (Cur) was then incorporated into SeNPs to produce selenium-curcumin nanoparticles. For surface modification, CS or folic acid-conjugated chitosan (Cs-FA) solutions were added dropwise to SeNP or selenium-curcumin suspensions, followed by centrifugation to complete coating formation. This process yielded four nanoparticle formulations: Se/Cs, Se@Cur/Cs, Se/Cs-FA, and Se@Cur/Cs-FA [61].

**Hyaluronic acid (HA):** Hyaluronic acid (HA) binds to CD44 receptors on tumor cells, enhancing active targeting and uptake in cancerous tissues. Zou et al. reported a novel method as described [62]. SeNPs were synthesized by mixing vitamin C and sodium selenite solutions under stirring at room temperature. HA was then added to form Hyaluronic acid (HA)-coated SeNPs. Paclitaxel dissolved in DMSO was incorporated into the HA-SeNPs solution, followed by stirring, dialysis, and centrifugation to obtain HA-SeNPs-Paclitaxel nanoparticles. A similar method was used to prepare coumarin-6-labeled Hyaluronic acid (HA)-SeNPs-Paclitaxel. Paclitaxel loading content and efficiency were determined using HPLC based on a calibration curve.

**Glucose:** Glucose is exploited for glucose transporter (GLUT)-mediated uptake in cancer cells, which have elevated glucose metabolism. In this approach, sodium selenite was combined with glucose solutions of varying concentrations and subjected to a brief autoclave treatment, during which selenium was reduced and assembled into nanoparticles. After the reaction mixture was cooled and depressurized, the SeNPs were isolated, washed multiple times to remove residual components, and freeze-dried [63].

##### Proteins and Antibodies (for Receptor-Mediated Targeting)

**Albumin (BSA, HSA):** Albumin improves stability and drug-binding capacity [64]. Albumin-coated SeNPs demonstrate enhanced biocompatibility and a longer circulation half-life. Xu et al. reported a method that was inspired by the self-assembly behavior of albumin, which can re-form nanoparticles through a denaturation-renaturation process. This study used denatured human serum albumin (HSA) and nano-selenium (Se) to fabricate HSA/Se nanoparticles via cystine-mediated reduction of sodium selenite. In this design, albumin (typically BSA) serves not only to control nanoparticle size, but also functions as both a size-regulating agent and as an encapsulating carrier during nano-Se formation [65].

**Monoclonal antibodies (e.g., anti-HER2, anti-EGFR):** Monoclonal antibodies enable highly specific targeting of cancer cells that overexpress those receptors. Antibody-functionalized SeNPs facilitate receptor-mediated endocytosis and precision drug delivery. Du et al. engineered a targeted delivery platform by creating trastuzumab-modified, BSA-coated, pyrotinib-loaded hollow mesoporous SeNPs (HBHSEP). The hollow mesoporous architecture was obtained using a silica-template method, in which selenium was deposited onto aminated silica particles, and the template was removed to form hollow SeNPs. Subsequent functionalization involved BSA coating to enhance stability, drug loading with pyrotinib, and trastuzumab conjugation via NHS/EDC chemistry, resulting in an HER2-targeted nanocarrier [66].

In addition, Huang et al. fabricated uniform and stable SeNPs (Se-5-Fu-Gd-P(Cet/YI-12)) with epidermal growth factor receptor (EGFR)-targeting and tumor microenvironment-responsive properties [13]. The system was designed using EGFR as the targeting ligand, gadolinium chelate as a magnetic resonance imaging (MRI) contrast agent, and 5-fluorouracil (5-Fu) together with cetuximab as therapeutic payloads. Polyamidoamine (PAMAM) and 3,3′-dithiobis(sulfosuccinimidyl propionate) served as responsive linkers sensitive to intratumoral glutathione and pH, enabling both treatment and diagnosis of nasopharyngeal carcinoma (NPC).

##### Other Functional Coatings

Table 1 summarizes the general applications and advantages of different ligand classes that were used for SeNPs. Plant-derived ligands (polyphenols, flavonoids) like tannic acid or catechin contribute antioxidant and antimicrobial effects while stabilizing the nanoparticles during green synthesis [67,68]. SeNPs can be synthesized biologically using living organisms such as plants, algae, fungi, and bacteria. Various biological sources and synthesis pathways have been explored for SeNP production [69,70]. The growing preference for these biological methods stems from their eco-friendly nature, which addresses limitations associated with conventional chemical and physical synthesis techniques, such as high cost, operational complexity, and potential toxicity, making them a safer and more sustainable alternative for SeNPs fabrication [71].

Another great example will be the report by Gulbay et al., where they used a phytochemical compound found in the plant Nigella sativa named “Thymoquinone” as a ligand to SeNPs. Thymoquinone (TQ)-encapsulated SeNPs(TQ-SeNPs) were prepared via a redox reaction between sodium selenite and ascorbic acid, with thymoquinone (TQ) as a stabilizer. The reaction mixture was stirred at 30 °C in the dark for 4 h, followed by 48 h of dialysis to remove excess reagents. The purified product was centrifuged and freeze-dried to obtain TQ-SeNPs [72]. This method was an extension of previously reported ones [47,73].

Recently, Liu and co-workers developed a facile procedure for the preparation of 5-fluorouracil surface-decorated SeNPs with improved anticancer activity. It was found that the surface functionalization of spherical SeNPs with 5-fluorouracil through physical adsorption increased the uptake of the SeNPs by cells [74].

### 1.4. Key Physicochemical Properties of SeNP-Based Drug Delivery Systems

The physicochemical characteristics of SeNPs are decisive in determining their drug-loading capacity, stability, biological performance, and therapeutic outcomes. Parameters such as particle size, morphology, surface chemistry, and drug-loading efficiency influence biodistribution, pharmacokinetics, and cellular uptake. Therefore, a comprehensive understanding of these attributes is essential to engineering SeNPs as reliable and effective nanocarriers for precision drug delivery [75,76].

#### 1.4.1. Particle Size and Morphology

Particle size and its distribution are among the most critical determinants of SeNPs’ biological fate. Nevertheless, nano-scale dimensions (10 to 200 nm) enable enhanced permeability and retention in tumor tissues, which favors drug accumulation in diseased sites while maintaining minimal off-target toxicity. Smaller SeNPs (<100 nm) had a high surface-to-volume ratio that resulted in a much higher drug-loading potential and quicker uptake by cells. However, overly small nanoparticles may undergo rapid renal clearance, limiting systemic retention. Conversely, larger SeNPs (>200 nm) may induce aggregation and be more readily captured by the reticuloendothelial system (RES), reducing circulation time [77,78,79,80,81].

**Figure 3:**
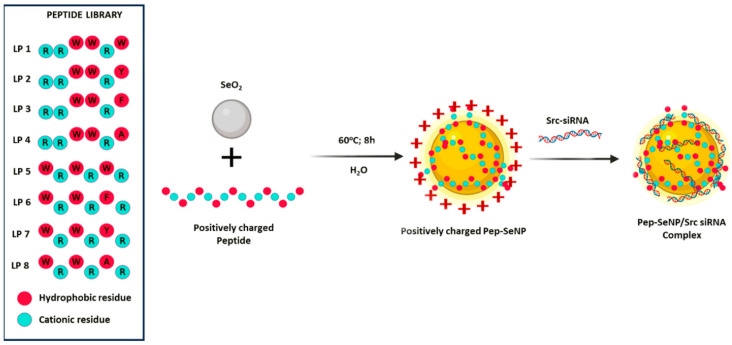
Schematic Representation of Synthesis of Peptide-Functionalized SeNPs and Formation of PepSeNPs/siRNA Complexes. This Figure was reprinted with permission from reference [58]. Copyright 2025 American Chemical Society.

For instance, peptide-containing arginine, tryptophan, and cysteine-capped SeNPs ([W_5_R_4_C]-SeNPs) were found to be in the size range of 110–150 nm after 1 day of incubation [57]. ([W_5_R_4_C]-SeNPs formed spherical nanostructures where SeNPs were surrounded with a layer of peptide. Positively charged arginines and hydrophobic tryptophans contributed to intermolecular interactions, resulting in a spherical morphology. In addition, Suryakanta et al. fabricated eight short linear peptides (LP) primarily composed of tryptophan and arginine residues, designed for the one-pot synthesis of peptide-capped SeNPs (LP-SeNPs) (Figure 3).

The synthesized Linear Peptide-SeNPs were characterized using field emission scanning electron microscopy (FE-SEM). The results showed that among the peptide-functionalized SeNPs, LP1-SeNP, LP3-SeNP, LP5-SeNP, LP6-SeNP, and LP7-SeNP predominantly exhibited spherical morphologies, whereas LP2-SeNP displayed a polygonal shape. In contrast, LP4-SeNP appeared highly irregular, and LP8-SeNP showed an oval morphology (Figure 3). Analysis of nanoparticles showed that the size dispersion and average particle diameter varied. Whereas LP5-SeNP and LP7-SeNP were monodisperse, 48 nm and 68 nm in size, correspondingly, the other LP-SeNPs were polydisperse, with concentration averages of 175, 270, 200, 64, 230, and 225 nm for LP1-SeNP, LP2-SeNP, LP3-SeNP, LP4-SeNP, LP6-SeNP, and LP8-SeNP, respectively. This may be that the LP5 peptide, with alternating tryptophan and arginine residues, optimized the particle growth mode, resulting in nanoscale-sized particles. The alternating arrangement of hydrophobic and cationic amino acids appears to play a critical role in both the synthesis and stabilization of SeNPs. This observation aligns with previous studies, which report that the free amino group in the indole ring of tryptophan can reduce metal ions, facilitating nanoparticle formation [58].

In another study, Li et al. reported the synthesis of polyethylenimine (PEI)-modified SeNPs (Se@PEI@siRNA) for cellular delivery of siRNA [82]. The particles were found to be in circular-shaped morphologies with an average size of ~50 nm as revealed by Transmission Electron Microscopy (TEM).

The use of SeNPs for delivering siRNA was extended by Xia et al. They fabricated biocompatible SeNPs loaded with RGDfC to be employed as a tumor-targeting gene delivery vehicle, namely RGDfC-SeNPs. TEM revealed that the RGDfC-SeNPs consisted of uniformly spherical particles with an average diameter of approximately 75 nm. Elemental analysis detected a distinct selenium signal corresponding to the nanoparticles, along with carbon (C) and oxygen (O) signals derived from the RGDfC peptide in the spectrum of RGDfC-Se@siRNA, confirming the successful conjugation of RGDfC onto the surface of the SeNPs. The size distribution of RGDfC-SeNPs both in water and phosphate-buffered saline revealed that RGDfC-SeNPs remained stable, with a size of less than 150 nm, which did not change over time. In other words, RGDfC-SeNPs were revealed to be extremely stable in aqueous and PBS environments [83].

Uniform particle size distribution ensures predictable pharmacokinetics and consistent therapeutic outcomes. Polydispersity index (PDI) values below 0.3 are generally indicative of monodisperse systems, which are suitable for biomedical applications. Several synthesis methods, including green reduction using polysaccharides or amino acids, or chemical reduction employing stabilizers, have been optimized to achieve narrow size distributions. For example, chitosan- or BSA-stabilized SeNPs often display sizes between 50 and 150 nm with improved colloidal stability and controlled drug release profiles. Thus, precise control over particle size and homogeneity is crucial for enhancing SeNPs’ clinical performance [84,85,86].

The morphology of SeNPs significantly influences their physicochemical and biological interactions. While spherical nanoparticles are most common due to their thermodynamic stability and ease of synthesis, other morphologies, such as rod-shaped, cubic, or flower-like SeNPs, have been reported to alter cellular uptake pathways and drug release kinetics. Spherical SeNPs have a relatively uniform surface energy distribution and are therefore more predictable in biological media, while anisotropic SeNP forms exhibit high surface reactivity and, in the case of drug delivery, more efficient drug adsorption [84,87,88,89].

Moreover, the shape affects interaction with cellular membranes and endocytic mechanisms. Studies have shown that rod-like or elongated SeNPs may penetrate cells more efficiently via clathrin-mediated pathways, while spherical SeNPs favor caveolae-mediated uptake [90]. Morphological tuning can thus be exploited to optimize delivery to specific tissues or organelles. Characterization techniques such as TEM, scanning electron microscopy (SEM), and atomic force microscopy (AFM) are routinely used to assess SeNP morphology and correlate it with biological performance [76,91].

As described above, SeNPs can be directly functionalized with anticancer drugs, such as 5-FU, to form 5-FU-SeNPs, which exhibit enhanced anticancer efficacy [74]. The morphology and chemical composition of 5-FU-SeNPs were characterized using various spectroscopic and microscopic methods. TEM images of SeNPs in the absence and presence of the capping agent 5-FU revealed distinct morphological differences. The 5-FU-SeNPs exhibited a monodisperse and uniform spherical structure with an average diameter of approximately 70 nm. In contrast, uncapped SeNPs in aqueous solution showed severe aggregation due to their high surface energy, leading to noticeable precipitation.

Another successful example of drug binding SeNPs was β-cyclodextrin (β-CD)-folate (FA)-modified SeNPs, which were further loaded with PTX (Se@β-CD-FA@PTX). The TEM images of particles showed that the average diameter of the nanoparticles was found to be around 70 nm. The nanoparticles were observed to be monodisperse and spherical in shape [14].

#### 1.4.2. Surface Functionalization and Encapsulation

The role of surface functionalization was discussed thoroughly in Section 1.3.1, “Ligands,” where various ligands can bind to SeNPs, thereby determining their application.

**Figure 4:**
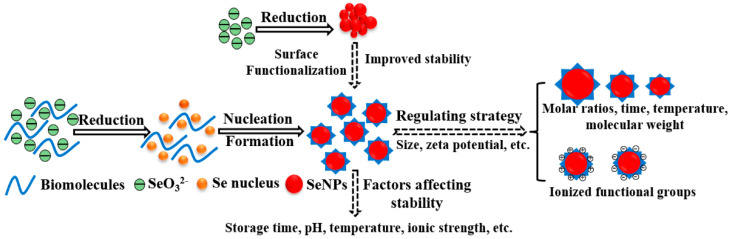
Schematic diagram of the formation process of biomolecule-functionalized SeNPs. This Figure was reproduced from reference [92] by Chen et al. under the terms of the Creative Commons Attribution (CC BY) license.

Surface chemistry is the single most flexible lever for improving SeNP performance. Bare SeNPs are prone to aggregation and rapid opsonization; therefore, surface modification is used to (a) stabilize colloids, (b) prolong circulation, (c) target specific cells/tissues, and (d) enable stimuli-responsive release (Figure 4) [92].

Encapsulation of SeNPs into liposomes, polymeric micelles, or mesoporous silica shells can combine the intrinsic bioactivity of selenium with enhanced payload protection and controlled release profiles (e.g., SeNP-loaded liposomes or SeNP@MSNs). These hybrid systems often show improved stability and multifunctionality [93,94]. This means that the liposomes, polymeric micelles, or mesoporous silica shells protect the SeNPs from degradation in physiological conditions, at the same time, control the release process of SeNPs or loaded cargos. This combination would provide natural biological activity of selenium with the advantage of advanced delivery carriers. For instance, Asadizadeh et al. reported that SeNPs were encapsulated using the thin-film hydration method, yielding liposomal formulations with an average hydrodynamic diameter of 235 ± 12 nm, a polydispersity index (PDI) of 0.15 ± 0.02, and a zeta potential of approximately −28.6 ± 1.7 mV, indicating decent colloidal stability and narrow size distribution.

Selmani et al. took advantage of thiolated chitosan. SeNP-loaded liposomes were prepared through a single-step microfluidics-assisted chemical reduction and assembly process. Following liposome formation, chitosan-*N*-acetylcysteine was covalently attached to the Lip-SeNPs. The resulting Lip-SeNPs were characterized in terms of composition, morphology, particle size, zeta potential, lipid organization, loading efficiency, and radical scavenging activity. The thiolated Lip-SeNPs exhibited a positive surface charge and an average size of approximately 250 nm, demonstrating strong adhesion to the mucus layer without entering the enterocytes. This approach allowed for the simultaneous chemical reduction of selenite (Na_2_SeO_3_) to elemental selenium, the formation of SeNPs, the assembly of liposomes, and the encapsulation of SeNPs within the liposomes, all in a single-step process [95].

Surface functionalization of SeNPs using amino acids containing a sulfur moiety is another strategy that was used by Shirazi et al. Peptide [W_5_R_4_C] combined multiple functional residues with distinct roles in nanoparticle formation and stabilization. The hydrophobic tryptophan residues likely promoted self-assembly and facilitated the formation of peptide-coated SeNPs through hydrophobic interactions. Meanwhile, the cysteine residue may have contributed to the resulting morphology by forming strong interactions with the SeNP surface. The thiol (–SH) group of cysteine can stabilize selenium within the chemical environment, possibly through the formation of selenium-sulfur covalent bonds between the Se atoms of the nanoparticles and the thiol groups of the peptide [57].

**Figure 5:**
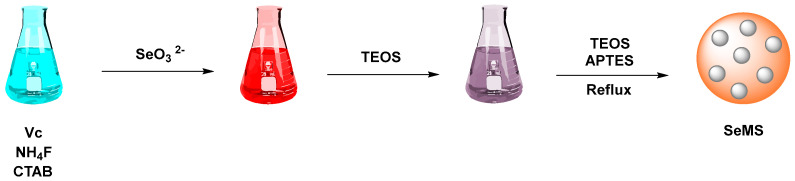
An illustration of the synthesis of SeNPs functionalized with mesoporous silica.

Another method to functionalize SeNPs by mesoporous silica-coated HA was used by He et al. [86]. The SeNPs were surface-coated with mesoporous silica. Cetyltrimethylammonium bromide (CTAB), ascorbic acid, and ammonium fluoride were dissolved in water and stirred at an elevated temperature. Sodium selenite was then added dropwise to form SeNPs, followed by the gradual addition of Tetraethyl orthosilicate (Si(OC_2_H_5_)_4_) TEOS to create a silica coating. Subsequent functionalization and reflux steps followed the same procedure as MSN synthesis (Figure 5).

#### 1.4.3. Cargo Loading

Cargo loading on SeNP platforms can be accomplished by several strategies: physical adsorption, electrostatic complexation, encapsulation (within shells or composite carriers), and covalent conjugation. Optimizing the loading method depends on the drug’s physicochemical properties (hydrophobicity vs. hydrophilicity, molecular weight, and ionic character) and the intended release mechanism.

##### Common Loading Strategies and Their Implications

Physical adsorption and hydrophobic interactions between SeNPs and the cargo molecule or drug are likely the most impactful elements to handle the binding process. Small hydrophobic drugs adsorb onto hydrophobic domains of a capped SeNP or within polymer shells. Adsorption is a simple and straightforward method; however, it may lead to premature drug desorption under physiological conditions. To minimize burst release, stabilizing agents or secondary encapsulation techniques are commonly employed. One instance is the use of tryptophan in peptide structures that create hydrophobic pockets and facilitate cargo encapsulation [57,58]. This process was performed with several anticancer drugs to confirm the outcomes. The antiproliferative effects of various anticancer drugs, doxorubicin, gemcitabine, clofarabine, etoposide, camptothecin, irinotecan, epirubicin, fludarabine, dasatinib, and paclitaxel, were enhanced in the presence of [W_5_R_4_C]–SeNPs (50 μM), showing respective increases of 38%, 49%, 36%, 36%, 31%, 30%, 30%, 28%, 24%, and 17% after 48 h of incubation in SK-OV-3 cells.

Electrostatic complexation is an initial step of several drug encapsulation processes. Charged drugs (e.g., nucleic acids, certain small molecules) are complexed to oppositely charged polymer-coated SeNPs (e.g., chitosan or PEI coatings). This method is widely used for gene/RNA delivery, allowing for relatively high loading efficiencies while enabling controlled release through competitive ionic exchange or pH shifts [95,96].

Covalent conjugation is another method to bind SeNPs to a drug or a cargo molecule. Covalent attachment reduces premature leakage and can improve pharmacokinetic predictability but may require careful linker design to ensure bioactivity is restored upon release. For instance, Ahmed et al. synthesized colistin-conjugated SeNPs (Col-SeNPs) as a dual-function nano-sized therapeutic agent against multidrug-resistant *Pseudomonas aeruginosa*, antifungal-drug-resistant *Candida* spp., and human breast carcinoma (MCF-7) cells [97]. Based on the protocol, SeNPs were dispersed in distilled water using sonication for 10 min. A colistin solution was added dropwise, and the mixture was incubated with shaking for 24 h at 25 °C. After centrifugation, the sediment was collected, completing the coating process designed to enhance antimicrobial activity [98].

## 2. Biological Properties

### 2.1. SeNPs in Clinical Trials

SeNPs have been proposed for use in various biological applications. Clinicians have tried to utilize them for the treatment of different types of disease states. For instance, Rezaeimanesh et al. reported a triple-blind randomized clinical trial in 60 patients with multiple sclerosis, which evaluated the effects of crocin–selenium nanoparticles (Cor@SeNs) on cognitive performance and oxidative stress. Over 12 weeks, participants received either Cor@SeNs (5.74 mg crocin + 55 µg selenium) or a placebo. The intervention produced a significant increase in total antioxidant capacity compared with placebo and showed significant time-related improvements in several cognitive tests, including CVLT-II and SDMT, in both groups. Although the Cor@SeNs group demonstrated greater improvements in cognitive outcomes and antioxidant markers, these differences were not statistically significant. As the first clinical trial assessing this nanoformulation at low doses, the study suggests potential benefits. There is a need for longer and higher-dose investigations to clarify its therapeutic value [99]

In another study, Noormohammadi et al. conducted a triple-blind, randomized, placebo-controlled trial to evaluate whether nano-selenium supplementation (55 µg/day for 12 weeks) could modulate the expression of JAK2, STAT3, and IDO1 in patients with major depressive disorder receiving standard sertraline therapy. Among the 50 participants, both the nano-selenium and placebo groups showed significant reductions in JAK2 and STAT3 expressions, with slightly greater decreases in the nano-selenium group; however, these between-group differences were not statistically significant. As the first investigation exploring nano-selenium as an adjunct intervention for major depressive disorder, the results suggest potentially meaningful molecular effects while clearly indicating that further research is required to assess its clinical relevance [100].

In another investigation, Noormohammadi et al. conducted a randomized, triple-blind, placebo-controlled trial evaluating nano-selenium (55 µg/day) as an adjunct to sertraline in adults newly diagnosed with major depressive disorder. Among the 42 participants who completed the 12-week study, nano-selenium significantly reduced depressive symptoms and improved antioxidant status, demonstrated by increases in total antioxidant capacity and glutathione peroxidase levels. Although malondialdehyde levels decreased in both groups, the between-group difference was not statistically significant. Overall, the results suggest that nano-selenium may serve as a safe and beneficial adjunct therapy for MDD; however, the study’s modest sample size and short duration require larger, longer-term clinical trials [101].

In general, clinical evaluation of SeNPs remains extremely limited, with only a small number of human trials, primarily in major depressive disorder (MDD) and multiple sclerosis (MS), reported to date. These studies are modest in scale, typically involving around 50–60 participants, and are relatively short in duration, often spanning just 12 weeks. Additionally, the administered selenium doses are low (approximately 55 µg/day), which may restrict the applicability of current findings to therapeutic areas that require higher or more cytotoxic concentrations, such as oncology. Although no major safety concerns were identified in these early investigations, the long-term safety profile of SeNPs in humans remains largely unknown, particularly given that preclinical studies highlight potential risks associated with dose, particle size, and exposure duration. Furthermore, significant regulatory and translational barriers remain, as the complexity of nanoparticle systems, encompassing factors such as stability, surface chemistry, and biodistribution, poses additional challenges compared to traditional small-molecule therapeutics.

### 2.2. Mechanism of Cellular Drug Delivery

Drugs or Molecular cargos can enter cells through several pathways, including micropinocytosis, phagocytosis, and various forms of receptor-mediated endocytosis (RME). RME may proceed via caveolae-mediated, clathrin-mediated, or clathrin/caveolae-independent endocytic routes [102].

SeNPs exhibit multiple cellular penetration mechanisms due to their tunable size, surface charge, and ease of functionalization. Their uptake typically proceeds via energy-dependent endocytosis, although passive mechanisms also contribute under certain conditions [90].

Shirazi et al. conducted detailed mechanistic studies to better elucidate how these nanoparticles are internalized by cells [58]. Their findings showed that the uptake of cargo-loaded [W_5_R_4_C]-SeNPs in SK-OV-3 cells was not significantly reduced after 2 h of incubation at 37 °C in the presence of chloroquine, chlorpromazine, or methyl-β-cyclodextrin. This indicates that clathrin-mediated endocytosis [103,104], caveolae-mediated endocytosis, and phagocytosis are not the sole pathways involved in cellular uptake. However, treatment with nystatin and EIA reduced cargo uptake by 52% and 40%, respectively, suggesting that caveolae-mediated endocytosis and macropinocytosis contribute to the internalization of functionalized SeNPs. Furthermore, sodium azide, used as an ATP-depleting agent, did not affect cargo uptake, indicating that the internalization process is not dependent on ATP [105].

In another investigation, Fang et al. engineered a multifunctional SeNP system for the treatment of HBV-associated hepatocellular carcinoma. The SeNPs were surface-functionalized with baicalin and folic acid (FA), which enhanced their bioactivity, cellular permeability, and tumor-targeting capacity. The resulting nano formulation exhibited high specificity toward HepG2.2.15 cells, preferentially accumulating in lysosomes and entering cells primarily through caveolae-mediated endocytosis. Additionally, B–SeNPs–FA suppressed ROS generation and downregulated key proliferation-related genes, ultimately inducing cytotoxicity in HBV-infected liver cancer cells. These tailored SeNPs represent a promising therapeutic platform for HBV-related liver cancer [106].

In addition, Chen et al. compiled a comprehensive Table summarizing various ligand-conjugated SeNPs, their associated endocytic pathways, and proposed biochemical mechanisms [92]. Table 2 presents a modified version of their data.

## 3. Challenges and Limitations

Although SeNPs hold remarkable promises as multifunctional nanocarriers, several physicochemical and biological limitations still hinder their clinical translation. These challenges arise primarily from issues of stability, toxicity, large-scale synthesis, and regulatory compliance. Understanding and addressing these constraints is crucial to ensure reproducibility, safety, and therapeutic efficacy in clinical settings [75,114].

### 3.1. Stability in Biological Environments

One of the foremost challenges in SeNP-based drug delivery systems is maintaining colloidal stability in physiological environments. Bare SeNPs are prone to aggregation, oxidation, and dissolution, particularly under high ionic strength or protein-rich conditions such as blood plasma [76]. Instability can alter particle size and surface charge, thereby modifying pharmacokinetics and reducing bioavailability. Moreover, oxidation of elemental selenium (Se^0^) to selenite (Se^4+^) or selenate (Se^6+^) may produce reactive oxygen species (ROS), compromise redox homeostasis, and increase cytotoxicity [85].

Surface functionalization with biocompatible polymers, such as polyethylene glycol (PEG), chitosan, or albumin, has proven effective in improving aqueous stability and preventing aggregation [65,114]. However, these coatings sometimes mask functional groups vital for drug loading or receptor targeting. The other major issue is the ability to provide long-term storage. SeNPs start to merge and grow through Ostwald ripening over time, particularly in high humidity or temperature. Although encapsulation in lipid or polymeric matrices can solve this problem, developability or production cost is compromised [91].

### 3.2. Immunogenicity and Toxicity Concerns

While selenium is an essential micronutrient, its narrow therapeutic window poses considerable safety challenges. The fine line between antioxidant protection and pro-oxidant toxicity is highly dose-dependent, and nanoparticulate selenium may exhibit unexpected biological reactivity due to its high surface area [75,115]. Uncoated or poorly stabilized SeNPs can trigger oxidative stress, mitochondrial dysfunction, or DNA damage in normal tissues. In vivo studies have demonstrated dose-related hepatotoxicity, nephrotoxicity, and neurotoxicity at elevated concentrations [86].

Immunogenicity represents another critical limitation. Upon systemic administration, SeNPs may be recognized as foreign bodies by the mononuclear phagocyte system (MPS), leading to opsonization, macrophage uptake, and accelerated clearance [76]. Functionalization with hydrophilic polymers or biomimetic coatings, such as erythrocyte membranes, can reduce immune recognition; however, these strategies complicate synthesis and raise concerns about reproducibility. Moreover, batch-dependent variability in surface chemistry can lead to inconsistent immune responses and unpredictable biodistribution. Therefore, achieving a balance between biocompatibility, biodegradability, and immune tolerance remains a central challenge in the clinical translation of SeNPs.

### 3.3. Scalability and Reproducibility

From a manufacturing standpoint, the reproducible large-scale synthesis of SeNPs is still a formidable barrier. Many current laboratory methods, such as chemical reduction or green synthesis, are highly sensitive to reaction parameters, including temperature, pH, precursor concentration, and reductant ratios [76,86]. Small deviations can drastically alter nanoparticle size, morphology, or surface chemistry, thereby affecting biological behavior and therapeutic efficacy.

Additionally, while eco-friendly, biogenic synthesis routes often lack batch-to-batch consistency due to the inherent inconsistency of the biological extract or microbial culture. Commercial industrialization requires control of contamination, residual reagents, and endotoxins, which compromise biocompatibility. An emerging solution to this problem is microfluidics and continuous flow synthesis systems that offer high precision, automation, and scalability. However, such systems also require specialized infrastructure and increase production costs, thereby reducing their capabilities in early-stage pharmaceutical development [99]. However, such systems require specialized infrastructure and incur high production costs, which may limit their adoption in the early stages of pharmaceutical development.

Reproducibility also extends to surface functionalization and drug-loading efficiency. Achieving uniform ligand density and consistent drug payloads remains a technically demanding task. These challenges underscore the need for standardized protocols and validated analytical tools to ensure batch-to-batch consistency in clinical-grade SeNP formulations [91].

### 3.4. Regulatory and Translational Barriers

Despite extensive preclinical evidence supporting the therapeutic benefits of SeNPs, regulatory approval and clinical translation remain in their infancy. The lack of clear guidelines for SeNP-based materials creates difficulty in classifying them as drugs, dietary supplements, or regular nanomedicines. The FDA and the EMA expect comprehensive toxicokinetic, genotoxic, and immunotoxicological data, and standardized nanoparticles have not yet been established.

However, the most significant barrier is the absence of long-term safety data. As a result of short-term biocompatibility, the long-term or cumulative effects of chronic exposure are still largely unresolved. Synthesis and subsequent functionalization are carried out, making it impossible to establish universal quality requirements. Furthermore, the versatility of synthesis and functionalization strategies makes it challenging to determine quality improvements that are more direct and beneficial.

Looking forward, the development of Good Manufacturing Practice guidelines for SeNPs and more robust in vivo biodistribution and metabolism studies are crucial in bringing laboratory inventions to real-world therapy. Without these critical aspects, SeNP-based modalities are unlikely to progress beyond experimental or nutraceutical niches, as they will not achieve clinical justification.

## 4. Future Perspectives

The rapidly growing field of SeNP-based drug delivery has emerged over the last decade from proof-of-concept studies to highly complex multimodal systems capable of targeted, stimuli-responsive therapy. At the same time, the clinical translation of SeNPs from laboratory discovery to clinical realization remains a significant challenge. The future agenda should therefore include increasing inherent biological knowledge, defining material standards, and producing current clinical protocols that more efficiently realize the therapeutic potential of these novel nanocarriers.

### 4.1. Toward Precision and Personalized Nanomedicine

The subsequent generation of SeNP-based systems would undoubtedly be aimed far more firmly at individualized treatment. No one fights a war with the same assets, and a new age of personalized physiology-guided nanoparticle style and dosing modification will quickly emerge. Prior to this, notions such as computational modeling, smart learning, and the utilization of artificial intelligence may provide this predictive control over nanomaterials-related biological dynamics, permitting the rational design of particle size, geometry, and surfactant changes in preferences. In addition, the use of molecular profiling information might allow this individualization feature to be utilized to disrupt the Redox equilibrium characteristic of disease conditions such as cancer, neurodegeneration, or prolonged inflammation [65,86].

### 4.2. Engineering Smart and Hybrid Selenium Systems

Emerging research is focusing on hybrid and stimuli-responsive SeNPs that combine the antioxidant, redox-active nature of selenium with structural and functional components from polymers, lipids, or biomimetic coatings. These multifunctional systems can respond dynamically to biological cues, such as pH, temperature, or glutathione concentration, allowing precise spatiotemporal drug release [85,86]. The integration of SeNPs into lipid-based carriers, polymeric hydrogels, or magnetic nanocomposites further expands their functionality for imaging, photothermal therapy, and controlled release.

A particularly promising avenue is the development of biomimetic SeNPs cloaked with natural cell membranes (e.g., erythrocyte or cancer cell membranes). These coatings provide immune systems, prolonged circulation, and active homing toward disease sites [116]. Moreover, hybrid SeNPs that incorporate trace metals (e.g., Au–Se, Fe–Se) or semiconductor components can exhibit synergistic redox and photothermal properties, enhancing therapeutic versatility while maintaining selenium’s intrinsic biological activity.

### 4.3. Integrating Regulatory, Safety, and Clinical Frameworks

The long-term success of SeNPs in drug delivery will depend not only on technological innovation but also on regulatory alignment and clinical validation. Establishing a harmonized regulatory framework for nanomedicine, specifically addressing selenium-based materials, is a priority [117]. Regulatory agencies must establish clear criteria for evaluating the pharmacokinetics, toxicity, immunogenicity, and biodegradation of nanoparticles.

Future preclinical studies should employ standardized animal models and longitudinal designs to assess chronic toxicity and clearance pathways. Advanced imaging and metabolomics approaches could track selenium biodistribution and transformation in vivo, providing essential safety data [75]. Furthermore, integrating pharmacoeconomic analyses early in the development pipeline will help identify clinically viable applications that balance innovation with affordability.

Public-private partnerships and cross-disciplinary collaborations will be crucial to advancing SeNPs from the bench to the bedside. By aligning material engineering, toxicology, and regulatory science, the field can progress toward clinically approved formulations for cancer therapy, neuroprotection, and anti-inflammatory treatment.

### 4.4. Outlook

In summary, several factors determine the future of SeNP-based drug delivery. They include precision design, regulatory readiness, and large-scale synthesis. Ongoing progress in bioengineering, computational modeling, and nanotoxicology indicates that SeNPs can transition from experimental carriers to clinically relevant therapeutic platforms. Their intrinsic redox activity, physicochemical tunability, and functionalization capacity render SeNPs highly promising for the future of nanomedicine. Nonetheless, clinical translation can only be achieved sustainably if initiatives to promote reproducibility, safety, and standardization are implemented worldwide. Provided that the current trend in interdisciplinary research continues, it is plausible that functionalized SeNPs will become the basis of redox-driven nanotherapeutics, offering safer, smarter, and more effective drug delivery solutions than before.

## 5. Conclusions

SeNPs have emerged as a unique class of nanomaterials due to their high potential for drug delivery, which is largely attributed to the unique physicochemical and biological properties of SeNPs. The most important unifying aspect of SeNP-based drug delivery systems throughout this evolution is the significant contribution of physicochemical parameters to biological performance. Conjugation of SeNPs with PEG, chitosan, peptides, antibodies, or small molecules improves not only the colloidal stability of SeNPs but also the ability to selectively interact with diseased tissues or cellular receptors. Simultaneously, the drug-loading capacity is also evolving, thanks to covalent conjugation, electrostatic interactions, and encapsulation, which enables the co-delivery of multiple agents for synergistic drug action. However, specific key challenges and limitations remain to be addressed before SeNPs can receive clinical approval in the drug delivery field.

Particularly, the stability in complex biological environments presents a challenge, as the redox-sensitive nature of selenium can result in oxidative degradation or aggregation under physiological conditions. Furthermore, the immunogenicity and dose-dependent toxicity of selenium must be thoroughly studied, as it has a narrow therapeutic range. Additionally, many scalability and reproducibility issues remain relevant for biological synthesis methods, where batch-to-batch variability is a common concern. Nonetheless, the broader future of SeNP-based systems will likely be determined by new directions in the field based on multidisciplinary approaches that will combine expertise in nanotechnology, pharmacology, and computational modeling. To this end, data-driven design, enabled by computational tools such as artificial intelligence and molecular dynamics, will be utilized to rationalize optimization of particle properties to improve biological predictability. Furthermore, the integration of SeNPs in hybrid or biomimetic systems, such as lipid nanoparticles or cell membrane-enveloped carriers, will broaden the range of their functions and add responsiveness to delivery.

## Figures and Tables

**Table 1:** Summary of ligand classes that are used in combination with SeNPs.

Ligand Category	Primary Purpose	Secondary Functions	Ref.
Polymers (PEG, chitosan, PLGA, PVP, alginate, silica)	Stabilization, colloidal stability	Control of drug release, stealth properties, and pH sensitivity	[53,54,55]
Peptides (RGD, Angiopep-2, RW-rich peptides)	Targeted delivery	Cell penetration, receptor binding	[57,58,59,60]
Small Molecules (FA, HA, glucose)	Receptor-specific targeting	Improved uptake, synergistic effect	[61,62,63]
Proteins/Antibodies (albumin, anti-HER2, anti-EGFR)	High-specificity targeting	Prolonged circulation, receptor-mediated uptake	[64,65,66]
Natural/Plant-derived ligands	Green synthesis & stabilization	Antioxidant or antimicrobial synergistic activity	[67,68]
Biological Sources	Biomimetic coating	Enhanced biocompatibility, lower toxicity	[71]

**Table 2:** List of ligands conjugated with SeNPs and their mechanism of cellular entry *.

Name of the Ligand-SeNPs	Mechanism of Entry	Ref.
Folic Acid-SeNPs	Clathrin and caveolin-mediated endocytosis	[107]
Poly-L-lysine-lactobionic acid- SeNPs	Asialoglycoprotein receptor-mediated endocytosis	[108]
Galactose-SeNPs	Clathrin-mediated endocytosis	[109]
*Gracilaria lemaneiformis* polysaccharide-SeNPs	αvβ3 integrin-mediated endocytosis	[110]
Lentinan-SeNPs	Caveolae-mediated endocytosis	[111]
Arginylglycylaspartic acid (RGD)-SeNPs	αvβ3 integrin-mediated endocytosis	[59]
GE11 Peptide-Se NPs	Lipid raft-mediated and clathrin-mediated endocytic pathway	[112]
human epidermal growth factor receptor 2 (HER2)-SeNPs	Receptor-mediated endocytosis	[113]

* This Table was modified from an original version that was published by Chen. et al., in Reference [90].

## Data Availability

No new data were created or analyzed in this study.

## Abbreviations

The following abbreviations are used in this manuscript:

AgNPs	Silver nanoparticles
SeNPs	Selenium nanoparticles
PEG	Polyethylene glycol
